# The Use of mHealth Apps for the Assessment and Management of Diabetes-Related Foot Health Outcomes: Systematic Review

**DOI:** 10.2196/47608

**Published:** 2023-10-04

**Authors:** Sean Sadler, James Gerrard, Angela Searle, Sean Lanting, Matthew West, Rhonda Wilson, Athula Ginige, Kerry Y Fang, Vivienne Chuter

**Affiliations:** 1 Western Sydney University Campbelltown Australia; 2 University of Newcastle Ourimbah Australia; 3 Central Australian Aboriginal Congress Mparntwe (Alice Springs) Australia; 4 University of Newcastle Gosford Australia; 5 Massey University Auckland New Zealand

**Keywords:** First Nations, Aboriginal, Torres Strait Islander, mobile health, mHealth, diabetes, diabetic, foot, systematic review, review methodology, mobile app, mobile apps, mobile phone

## Abstract

**Background:**

Globally, diabetes affects approximately 500 million people and is predicted to affect up to 700 million people by 2045. In Australia, the ongoing impact of colonization produces inequity in health care delivery and inequality in health care outcomes for First Nations Peoples, with diabetes rates 4 times those of non-Indigenous Australians. Evidence-based clinical practice has been shown to reduce complications of diabetes-related foot disease, including ulceration and amputation, by 50%. However, factors such as a lack of access to culturally safe care, geographical remoteness, and high costs associated with in-person care are key barriers for First Nations Peoples in accessing evidence-based care, leading to the development of innovative mobile health (mHealth) apps as a way to increase access to health services and improve knowledge and self-care management for people with diabetes.

**Objective:**

This study aims to evaluate studies investigating the use of mHealth apps for the assessment and management of diabetes-related foot health in First Nations Peoples in Australia and non-Indigenous populations globally.

**Methods:**

PubMed, Informit’s Indigenous Collection database, Ovid MEDLINE, Embase, CINAHL Complete, and Scopus were searched from inception to September 8, 2022. Hand searches of gray literature and reference lists of included studies were conducted. Studies describing mHealth apps developed for the assessment and management of diabetes-related foot health were eligible. Studies must include an evaluation (qualitative or quantitative) of the mHealth app. No language, publication date, or publication status restrictions were used. Quality appraisal was performed using the revised Cochrane risk-of-bias tool for randomized trials and the Health Evidence Bulletins Wales checklists for observational, cohort, and qualitative studies.

**Results:**

No studies specifically including First Nations Peoples in Australia were identified. Six studies in non-Indigenous populations with 361 participants were included. Foot care education was the main component of all mHealth apps. Of the 6 mHealth apps, 2 (33%) provided functionality for participants to enter health-related data; 1 (17%) included a messaging interface. The length of follow-up ranged from 1-6 months. Of the 6 studies, 1 (17%) reported high levels of acceptability of the mHealth app content for self-care by people with diabetes and diabetes specialists; the remaining 5 (83%) reported that participants had improved diabetes-related knowledge and self-management skills after using their mHealth app.

**Conclusions:**

The findings from this systematic review provide an overview of the features deployed in mHealth apps and indicate that this type of intervention can improve knowledge and self-care management skills in non-Indigenous people with diabetes. Future research needs to focus on mHealth apps for populations where there is inadequate or ineffective service delivery, including for First Nations Peoples and those living in geographically remote areas, as well as evaluate direct effects on diabetes-related foot disease outcomes.

**Trial Registration:**

PROSPERO CRD42022349087; https://tinyurl.com/35u6mmzd

## Introduction

### Background

The global diabetes prevalence rate for adults in 2021 was estimated to be >10.5% (n=536.6 million people), and it is expected to increase to 12.2% by 2045, with the associated health care expenditure costing an estimated US $1054 billion [1]. Diabetes-related foot disease (DFD) is a leading cause of disability worldwide [2]. It has a 5-year mortality rate and health care costs comparable with those of cancer [3], and it is associated with a low health-related quality of life [4]. The development of diabetes-related foot ulceration precedes up to 85% of nontraumatic amputations [5]. Diabetes-related complications in the lower limb, including peripheral neuropathy and peripheral arterial disease, typically precede the development of diabetes-related foot ulceration [6]. Collectively, these complications are a leading global cause of disability, hospitalization, and amputation [2]. In Australia, the ongoing effect of colonization has led to systemic racism, enduring disadvantage, trauma, political exclusion, and a health care system that is dismissive of Aboriginal health paradigms and world views [7,8]. This has perpetuated cultural safety deficits in Australian health care systems and contributed to disproportionately high rates of DFD, including a 4-fold increase in the risk of peripheral neuropathy and 5- and 6-fold increases in the risk of foot ulcer and amputation, respectively [9,10].

Evidence-based clinical practice, including the involvement of multidisciplinary teams and well-established care pathways, has been shown to reduce amputation rates in people with DFD by 50%, resulting in a significant reduction in patient and health care burden [11]. However, such care is not readily accessible to all patients [12,13]. It is well established that First Nations Peoples and non-Indigenous people living in rural and remote areas in Australia [14] and around the world [15-18] have worse overall health outcomes than their urban counterparts. This is due partly to the large geographical distance between health care services [19,20]. For First Nations Peoples in Australia, additional factors contributing to these outcomes include a lack of access to care that is culturally safe as well as a lack of workforce capacity, including difficulty in recruiting culturally capable practitioners [20,21]. This contributes to a system of care delivery that is frequently fragmented and inconsistent [22].

### Mobile Health Apps for Chronic Conditions

The use of app-based mobile health (mHealth) information systems is an emerging area of digital health that is increasingly being used to support the management of chronic conditions, including diabetes, particularly in rural and remote areas and across a variety of health systems [3]. mHealth apps are considered part of public health practice and have been shown to facilitate access to in-person local health services and other digital health technologies such as telehealth [23]. Studies have demonstrated that mHealth apps provide effective health education for lifestyle improvements such as smoking cessation and assist with self-monitoring for chronic disease [24]. A previous systematic review published in 2018 evaluated studies investigating mHealth apps for First Nations populations for any condition and found 9 studies in Australia, with the majority targeting mental health conditions (none for diabetes), with high rates of acceptability and engagement with the mHealth interventions by end users [25]. More broadly, studies in non-Indigenous populations internationally have demonstrated that mHealth apps are effective for improving diabetes-related knowledge and self-care management [26-28]. Because of the growing burden of DFD for First Nations Peoples in Australia and in the general population globally, a collective evaluation of studies investigating the use of mHealth apps designed to support diabetes-related foot health is required to help inform the design and implementation of future mHealth apps and assess their effectiveness for the assessment and management of diabetes.

Therefore, the aim of this systematic review was to identify the features of, and outcomes from using, mHealth apps for the assessment and management of diabetes-related foot health in First Nations Peoples in Australia and non-Indigenous populations globally.

## Methods

### Database Search

This review was conducted in accordance with the PRISMA (Preferred Reporting Items for Systematic Reviews and Meta-Analyses) [29] guidelines and registered with PROSPERO (CRD42022349087). An electronic database search of Ovid MEDLINE, Embase, CINAHL Complete, and Scopus was conducted from database inception to September 8, 2022 (Multimedia Appendix 1). At the same time, an additional search to identify any Australian First Nations–specific articles was conducted owing to the potential for some of this research to be published in First Nations–specific locations or gray literature. This consisted of an electronic database search of PubMed using specific Medical Subject Headings (MeSH) terms and Informit’s Indigenous Collection database (Multimedia Appendix 1). Differences in search terms and how terms were combined were adapted to the requirements of each database. Hand searches of gray literature were also conducted, and the sources included the Journal of the Australian Indigenous HealthInfoNet, Menzies School of Health Research, Services for Australian Rural and Remote Allied Health, and the Australian Institute of Health and Welfare. Reference lists of included studies and review articles were also searched. No language, publication date, or publication status restrictions were used.

### Inclusion and Exclusion Criteria

The inclusion criteria were studies describing mHealth apps developed for the management and assessment of DFD. The studies had to include some evaluation (qualitative or quantitative) of the mHealth app (which could include usability, acceptability, feasibility, or diabetes-related foot health outcomes). Studies were excluded if they only evaluated the technical design features of an mHealth app; if the mHealth app was designed for automatic detection of wounds or ulcers or for assessing the accuracy and reliability of wound images only; or if the mHealth app incorporated additional equipment such as thermal imaging cameras, pressure mats, activity monitors, pressure monitoring insoles, glucose monitors, smart socks, or foot temperature probes. Studies were also excluded if the mHealth app was delivered or assessed by people without diabetes or was for medical professionals only. The population, intervention, comparison, and outcomes (PICO) elements for this study are included in Multimedia Appendix 2.

Two authors (SS and AS) developed and pilot-tested the electronic searches, with 1 author (AS) conducting the search. Titles, abstracts, and full texts were assessed independently by 2 authors (JG and AS). Disagreements were resolved by consensus and a third author (SS) where necessary. Study data, including publication details (author, year, and location), participant characteristics (age, sex, and diabetes information), sample size, mHealth app features, and results, were extracted by 1 author (AS) and cross-checked by another author (SS). A descriptive synthesis of included study findings was performed.

### Quality Appraisal Tools

It was planned to use the Aboriginal and Torres Strait Islander Quality Appraisal Tool to assess studies that included First Nations Peoples from Australia [30]. The tool consists of 14 questions that are used to assess the cultural safety of the study. Answer options include “yes,” “partially,” “no,” and “unclear.” Broadly, questions relate to Community engagement; First Nations leadership and governance; intellectual and cultural rights; and translation of findings to policy, practice, and Community. To assess the potential risk of bias and methodological quality of the included studies, the following tools were used: the revised Cochrane risk-of-bias tool for randomized trials (RoB 2) [31] and the Health Evidence Bulletins Wales checklists for observational, cohort, and qualitative studies [32]. The RoB 2 tool assesses 5 areas of potential bias: randomization, deviations from the intended interventions, missing outcome data, measurement of the outcome, and the selection of the reported result. Each bias domain individually (and each study overall) is determined to display either a low or a high risk of bias or some concerns relating to the risk of bias. The observational study and qualitative study appraisal checklists designed by Health Evidence Bulletins Wales have been developed for a critical appraisal of observational and qualitative studies. These checklists were selected because the tools include a small number of key domains, are simple checklists rather than scales, and were developed using a variety of literature sources. The articles were rated independently by 2 authors (SS and AS), and any disagreements were resolved by consensus and a third author (VC) when necessary. There was no minimum level of quality required for inclusion in this review.

## Results

### Overview of Included Studies

**Figure 1:**
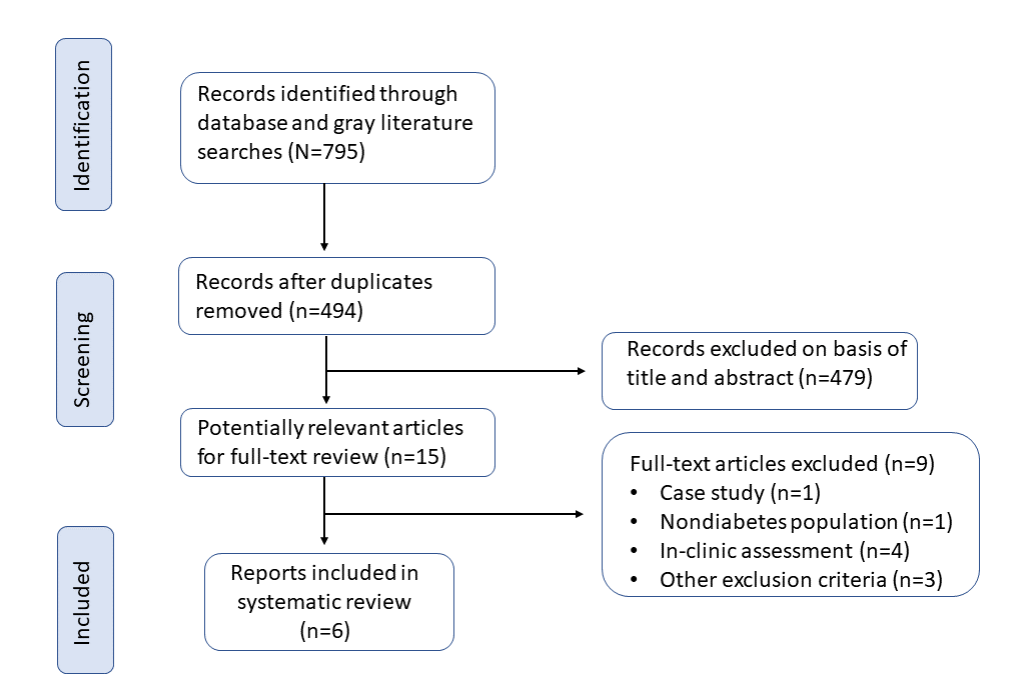
Flow diagram of study inclusion and exclusion.

The initial database search resulted in 494 citations (after the removal of duplicates), of which 15 (3%) were appropriate for full-text review (Figure 1). Of these 15 studies, we excluded 9 (60%) for the following reasons: mHealth app only being used in clinic with researchers (n=4, 44%) [33-36], wrong study type (n=1, 11%) [37], wrong study population (n=1, 11%) [38], design and development of the mHealth app only (n=1, 11%) [39], protocol (n=1, 11%) [40], and wound images only (n=1, 11%) [41] (Figure 1). No studies specific to First Nations Peoples from Australia were identified by this review. However, of the initial 494 studies, 6 (1.2%) that assessed mHealth apps for the management of diabetes-related foot health in non-Indigenous populations from Turkey [26,28], Brazil [27], Australia [42], Indonesia [43], and the Philippines [44] were included. The included studies, with sample sizes ranging from 20 to 130 people, included a total of 361 participants (Table 1). Of the 6 studies, 3 (50%) were randomized controlled trials [26,28,43], and the other 3 (50%) used a Delphi [27], qualitative [42], and pretest-posttest [44] methodology, respectively. The follow-up time of the studies ranged from 1 to 6 months. All studies were developed in an academic or university setting, with funding or financial support provided from external organizations for 5 (83%) [27,28,42-44] of the 6 studies.

**Table 1:** Overview of the included studies.

Study, year; country	Study design	Population	Description of app	Trial duration and assessments
Dincer and Bahçecik [26], 2021; Turkey	RCT^a^	Participants with DM^b^, without foot wounds, attending outpatient clinic at a university hospital N (female participants): control: 65 (33), intervention: 65 (31) Age (years), mean: control: 54.7 (SD 13.6), intervention: 49.5 (SD 17.4) DM duration (mo), median: control: 132 (range 1-540), intervention: 120 (range 1-504) Ulcer duration: NS^c^	Animation-supported Mobile Diabetic Foot Care Education app for use by individuals with type 2 DM, which consists of cartoon videos; push notifications twice weekly; and sections detailing (1) “Diabetes and Foot Problems,” (2) “Daily Foot Care,” (3) “What Kind of Socks?” (4) “What Kind of Shoes?” (5) “Nail Care,” and (6) “Things to Be Considered in Daily Life” Control group received one-off individual education in DM foot care in line with clinical guidelines at the hospital	1-mo at-home trial Patient assessment form Diabetes Foot Knowledge Questionnaire Diabetic Foot Care Self-Efficacy Scale Foot Self-Care Behavior Scale
Ferreira et al [27], 2019; Brazil	Develop and validate the content of the app	Participants with DM recruited by email and social media N (female participants): 20 (14) Age (years), median: 41.4 (range 21-65) DM duration (y), median: 14 (range 1-33) Ulcer duration: none	Free customized foot and ankle exercise software (internet and app) for people with DM; 3 main areas: foot care recommendations about DM and DPN^d^, self-assessment of feet (including FHSQ^e^, MNSI^f^, and falls occurrences), and customized exercises to increase strength and range of motion	Trial: 30-45 d, software use 2-3 times/wk Content validated using the Delphi methodology and a quantitative approach in 2 rounds with diabetes specialists (n=9) and participants with DM (n=20); reporting by descriptive statistics, absolute and relative frequencies, and the content validity index
Kilic and Karadağ [28], 2020; Turkey	Phase 1: pilot evaluation Phase 2: randomized feasibility trial	Phase 1 Convenience sample of participants with type 2 DM who visited the outpatient clinic N (female participants): 10 (NS) Age: NS DM duration: NS Ulcer duration: NS Phase 2 Convenience sample of participants with type 2 DM who visited the outpatient clinic N (female participants): control: 44 (20), intervention: 44 (29) Age (years), mean: control: 52.11 (SD 7.96), intervention: 51.16 (SD 8.27) DM duration (y), mean: control: 7.34 (SD 6.48), intervention: 7.36 (SD 5.43) Ulcer duration: none	Both the pilot and feasibility trials used the m-DAKBAS^g^ app, which consists of “Get information” and “Prevention” interfaces regarding DM foot education; a messaging interface to allow the administrator to send motivational and informative content and feedback regarding foot observations and blood glucose values; and participant data entry sections for (1) daily foot observations, (2) blood glucose levels, (3) photo sharing, and (4) a “Test yourself” interface All participants were given training about personal foot care and foot observations in the patient training room by the researcher The control group were given one-off training by the researcher through verbal instruction about the content of m-DAKBAS (definition of diabetic foot, risk factors, protective precautions, and daily foot care)	Phase 1 10 d at home No outcome measures reported but “some revisions to the app were made based on feedback” Phase 2 6 mo at home Diabetic foot knowledge form Foot Self-Care Behavior Scale Diabetic Foot Care Self-Efficacy Scale m-DAKBAS evaluation form Foot examination findings
Ogrin et al [42], 2018; Australia	Pilot study	Convenience sample of participants with DM attending community health center N (female participants): 33 (NS) Age (years), mean: 66.9 (SD 17.1) DM duration (y), mean: 17.1 (SD 10.3) Ulcer duration: NS	DM foot health education app Healthy Feet covering 3 main areas: daily foot care, consulting health providers, and education	12-wk trial Qualitative interviews
Pamungkas et al [43], 2022; Indonesia	RCT	Systematic random sampling of patients with DM at a community health center N (female patients): control: 30 (19), intervention: 30 (24) Age (years), mean: control: 54.50 (SD 9.20), intervention: 56.20 (SD 7.63) DM duration (y), mean: control: 7.34 (SD 6.48), intervention 7.36 (SD 5.43) Ulcer duration: none	DM coaching intervention consisting of narrative coaching (education), printed user guide, mindfulness coaching, skill-based coaching, brief app interaction, and reporting menu; the intervention also included foot care and DPN screening as well as telephone call and Zoom interaction with researcher to resolve problems and check progress Control group received routine services provided by community health centers	12-wk trial Diabetes Self-Management Questionnaire
Ridad et al [44], 2020; Philippines	1-group pre-post test	Purposive sampling of participants with DM at health clinic N (female participants): 30 (23) Age (years), range: 18-60 DM duration: NS Ulcer duration: NS	DiabEHT^h^ project consisting of (1) DiabEHT app (blood glucose monitoring tool; medication alarm; health education on diet, exercise, foot care, and oral hygiene; random notification; and data bank of blood glucose test results); (2) weekly support group activities and lecture series; and (3) the “diabooth inquiry station” at the health center that provides information, education, and communication materials and other free services such as blood pressure monitoring and blood glucose testing	6-week trial Summary of DM self-care activities

^a^RCT: randomized controlled trial.

^b^DM: diabetes mellitus.

^c^NS: not stated.

^d^DPN: diabetes-related peripheral neuropathy.

^e^FHSQ: Foot Health Status Questionnaire.

^f^MNSI: Michigan Neuropathy Screening Instrument.

^g^m-DAKBAS: Mobile Diabetic Foot Personal Care System.

^h^DiabEHT: Diabetic Electronic Health Tool.

### Components and Outcomes of the mHealth Apps

Foot care education was the main component of all mHealth apps included in this systematic review and covered topics such as daily foot care tasks, appropriate sock and shoe selection, nail care, and identification of peripheral neuropathy signs and symptoms (Table 1). Varied methods of education delivery were used by the studies, including DFD information pages (all studies), cartoon videos [26], test-yourself sections [28], mindfulness and skills coaching [43], 2D animation [27], and weekly support group activities and lectures [44]. Of the 6 mHealth apps, 2 (33%) provided functionality that allowed participants to enter health-related data [28,44], and 1 (17%) included a messaging interface [28].

Our systematic review has demonstrated short-term improvements in self-reported knowledge of, and self-care ability for, diabetes-related foot health with the use of an mHealth app. Specifically, of the 6 studies, 1 (17%) reported high levels of acceptability of the mHealth app content for self-care by people with diabetes and diabetes specialists [27] (Textbox 1), and the remaining 5 (83%) reported that participants had improved diabetes-related knowledge and self-management skills after using their mHealth app [26,28,42-44] (Textbox 1).

**Findings from the included studies.**

**Dincer and Bahçecik [26], 2021; Turkey**

- Animation-supported Mobile Diabetic Foot Care Education (M-DFCE) group had significantly higher knowledge, self-efficacy, and foot care behavior levels than the control group
  Diabetes Foot Knowledge Questionnaire: M-DFCE difference: mean 0.87 (SD 1.21) vs control difference: mean 0.01 (SD 1.25; *z*=−4.14; *P*<.01)
  Diabetic Foot Care Self-Efficacy Scale: M-DFCE difference: mean 13.4 (SD 25.4) vs control difference: mean 0.63 (SD 3.31; *z*=−3.05; *P*<.01)
  Foot Self-Care Behavior Scale: M-DFCE difference: mean 11.28 (SD 10.47) vs control difference: mean 0.6 (SD 24.85; *z*=−2.53; *P*<.05)
- Time using app during trial: not stated (NS)

**Ferreira et al [27], 2019; Brazil**

- Participants with diabetes mellitus: content validity index was 0.902 in the first round, and there was 97% approval from participants in the final round of the Delphi survey
- Time using app during trial: NS

**Kilic and Karadağ [28], 2020; Turkey**

- Poststudy knowledge scores were significantly higher in the experimental group (Mobile Diabetic Foot Personal Care System [m-DAKBAS] app) than in the control group
  Diabetic foot knowledge form: m-DAKBAS pretest score: mean 12.89 (SD 4.34) vs m-DAKBAS posttest score: mean 16.73 (SD 1.56; *P*<.01); control pretest score: mean 13.61 (SD 3.65) vs control posttest score: mean 15.05 (SD 2.17; *P*<.05); posttest scores were significantly different between control and intervention groups (*P*<.001)
  Foot Self-Care Behavior Scale: m-DAKBAS pretest score: mean 52.61 (SD 8.75) vs m-DAKBAS posttest score: mean 62.59 (SD 7.76; *P*<.01); control pretest score: mean 51.02 (SD 9.78) vs control posttest score: mean 59.45 (SD 10.53; *P*<.01); posttest scores were not significantly different between control and intervention groups (*P*=.23)
  Diabetic Foot Care Self-Efficacy Scale: m-DAKBAS pretest score: mean 65.59 (SD 17.4) vs m-DAKBAS posttest score: mean 74.16 (SD 13.46; *P*<.01); control pretest score: mean 65.59 (SD 16.68) vs control posttest score: mean 71.27 (SD 12.97; *P*<.05); posttest scores were not significantly different between control and intervention groups (*P*=.19)
  m-DAKBAS evaluation form: 93.2% would recommend the app, 93.2% believe it contributes to foot health, and 79.5% want to keep using it
- Foot examination
  Nonappropriate footwear: m-DAKBAS pretest assessment: n=14 vs m-DAKBAS posttest assessment: n=4 (*P*<.05); control pretest assessment: n=33 vs control posttest assessment: n=27
  Skin cracks: m-DAKBAS pretest assessment: n=18 vs m-DAKBAS posttest assessment: n=5 (*P*<.05); control pretest assessment: n=25 vs control posttest assessment: n=18
- Time using app during trial: NS

**Ogrin et al [42], 2018; Australia**

- App information would be highly useful for people newly diagnosed with diabetes or who had no previous exposure to foot care education
- Time using app during trial: median 16 (range 2-17) min/d over a median 4 (range 1-29) days
- Themes identified from qualitative data
  Personal context: this theme relates to the individuals’ perception of their knowledge and risk of serious foot complications
    Knowledge and self-care practices increased: “I think the app was good. I’ve got it downloaded now on my phone so I can read through it...every time I saw it...it reminded me...visually it stuck in my head and so I checked my feet. I moisturized my feet, I checked my nails, I made sure that my shoes were right...I did everything that I would normally know to do because this [study mobile phone] was like...a prompt.” [Patient 01]
    Already had the knowledge: “[B]ecause what I eventually saw on there, I already knew.” [Patient 02]
    Sufficient risk prevention measures were already in place: “We’ve had problems before and it happened so quickly. We’d go to either straight to the doctor or straight to the emergency, and so there’s absolutely nothing that it could tell me, that I don’t know already.” [Patient 03]
    App increased their awareness of foot complications: “I just—I didn’t know. I didn’t have an understanding of it. That was my—I knew you could get foot complications. But I never thought into that. Now, I know—you know if you’ve got a scratch you’ve got to be very aware. If you get a lump or something you’ve got to be—you and seek help straight away, so it doesn’t flow out of control.” [Patient 04]
    Behavior change needed only when a problem develops: “If you get something then you sort of think, oh, I should find out about that. But when you haven’t got it, you think, oh, that’s never going to happen to me.” [Patient 05]
    Other health issues: “When you’ve got celiac disease, on top of kidney problems, and then you’ve got diabetes, it just gets too hard, and you throw your hands in the air some nights.” [Patient 06]
  External context: this theme involved those aspects that had an impact on participants’ perceptions and ability to undertake self-care, beyond the personal context related to diabetes
    Carer responsibilities: “Well, as much as I know I should look in my shoes, I don’t. Every morning I get up, and my concern is getting my Dad out of the bed, because I’m a carer for him. I don’t have a lot of time for myself, and there’s no minute where I’m ever alone.” [Patient 07]
    Physical barriers: “Believe it or not, checking your feet is not a 1-person job. You have to have 2 people.” [Patient 08]
    Contradictory information: “I think I found from the moment that I got diagnosed with diabetes that one says one thing, another says the other, and in the end I just thought okay, I’m type 2.” [Patient 09]
  Educational preference: this theme involved participants having different preferred methods to receive information on foot health
    Varied use of smartphone features: “We use our mobile phones to make phone calls, and that’s virtually it. We’re not on the phones all the time or anything like that.” [Patient 03]; “I answer [the mobile phone], and if I don’t answer it, it goes to message bank. Then I collect it at the end of the day. That’s it. I don’t want to know any more about the phone. Everyone keeps raving about apps. I don’t know where they are.” [Patient 06]
    App-based education is good: “I actually liked the fact that it was on the smartphone because if I was out and wanted to have a quick look I could.” [Patient 07]
    Larger screen would make information easier to view: “You’re probably better to have that app on a tablet...Arms are not long enough, and the screen’s too small, and it’s very hard.” [Patient 10]
    Preference for other methods of information exchange: “Maybe if it can fit on 1 page, maybe you can put just the basic things on 1 page and like a magnet or something, we can stick on a fridge. So you don’t have to use the smartphone.” [Patient 11]; “It is and I really think that just to hand a person something like that and expect them to educate themselves I don’t think’s going to work. I don’t think it’s going to work. I don’t think there’s anything better than getting a group of people together and having the pictures on the screen and talking to them and educating the people that way.” [Patient 12]
  Content: this theme relates to the perception of participants on the content within the app
    The information was relevant and of interest: “I think the majority of it worked quite well. You could go into just 1 section and go through things. You didn’t have to go through the whole app because you’ve cross-referenced a number of the images and statements to various sections.” [Patient 13]
    Participants wanted varied levels of information: “I thought it was—look, it’s very basic.” [Patient 14]; “Some of the information was a bit too—I had to read it, and I think it was a little bit too hard...You need to break it down more simply.” [Patient 15]
    Preferred app to include all aspects of diabetes management, not just foot complications: “For me to be a hundred per cent on board with the whole—either foot check or test strip check, or whatever, I’d like to have an app that basically gives me all that on a daily basis. I would most probably go along and do that. But it’s having one for that, and then maybe another one for that, and all that. It just goes out the window for me.” [Patient 09]
    Needs more interaction: “To me, something like that would be a—maybe a checklist that could be customized. I guess if there were certain things that were indicated on the checklist, it could go further into that. I would see that as being a really useful thing where people—where you are prompted to do a check, where you pick the phone up or you get a reminder once a week.” [Patient 16]
  Target audience: this theme related to the participants’ thoughts on who would benefit most from the app
    Newly diagnosed individuals would benefit the most: “[I]f people are newly diagnosed, I think it’s a good starting point. I mean, it’s different for us because we’ve had it for a while...and we’ve had all the information. But if you’re newly diagnosed, I think it’s a great starting-off point.” [Patient 07]

**Pamungkas et al [43], 2022; Indonesia**

- Diabetes self-management (DSM) behaviors among the app group were improved compared with the control group in terms of dietary control, physical exercise, blood glucose monitoring, medication adherence, and screening of complications
- DSM screening results
  Dietary control: app group pretest score: mean 3.97 (SD 1.50) vs control pretest score: mean 4.37 (SD 1.27); app group posttest score: mean 8.83 (SD 1.80) vs control posttest score: mean 5.17 (SD 1.84; *P*<.05)
  Physical exercise: app group pretest score: mean 3.430 (SD 2.060) vs control pretest score: mean 3.230 (SD 1.466); app group posttest score: mean 6.870 (SD 1.360) vs control posttest score: mean 3.770 (SD 1.250; *P*<.01)
  Self-management of blood glucose: app group pretest score: mean 4.53 (SD 1.78) vs control pretest score: mean 4.07 (SD 1.57); app group posttest score: mean 10.23 (SD 1.48) vs control posttest score: mean 7.40 (SD 1.35; *P*<.01)
  Medication adherence: app group pretest score: mean 2.57 (SD 1.38) vs control pretest score: mean 3.03 (SD 1.79); app group posttest score: mean 4.97 (SD 1.00) vs control posttest score: mean 3.10 (SD 0.61; *P*<.01)
  Diabetes complications: app group pretest score: mean 1.90 (SD 2.07) vs control pretest score: mean 1.97 (SD 1.81); app group posttest score: mean 6.27 (SD 1.26) vs control posttest score: mean 2.13 (SD 1.17; *P*<.01)
- Clinical outcomes
  Glycated hemoglobin (HbA_1C_): app group pretest score: mean 8.04 (SD 1.96) vs control pretest score: mean 8.55 (SD 2.95); app group posttest score: mean 6.44 (SD 1.14) vs control posttest score: mean 8.24 (SD 2.61; *P*<.01)
  Systolic blood pressure: app group pretest score: mean 128.67 (SD 13.83) vs control pretest score: mean 128.33 (SD 18.21); app group posttest score: mean 120.00 (SD 11.14) vs control posttest score: mean 129.67 (SD 12.72; *P*<.01)
  Diastolic blood pressure: app group pretest score: mean 83.33 (SD 7.11) vs control pretest score: mean 82.00 (SD 8.87); app group posttest score: mean 72.50 (SD 8.69) vs control posttest score: mean 78.00 (SD 10.95; *P*<.05)
  BMI: app group pretest score: mean 23.70 (SD 3.53) vs control pretest score: mean 24.32 (SD 3.51); app group posttest score: mean 23.58 (SD 2.80) vs control posttest score: mean 24.28 (SD 2.69)
  High-density lipoproteins: app group pretest score: mean 65.17 (SD 14.41) vs control pretest score: mean 65.47 (SD 23.82); app group posttest score: mean 91.80 (SD 20.73) vs control posttest score: mean 61.57 (SD 19.35; *P*<.01)
  Low-density lipoproteins: app group pretest score: mean 117.63 (SD 49.61) vs control pretest score: mean 107.50 (SD 37.25); app group posttest score: mean 89.10 (SD 14.91) vs control posttest score: mean 109.57 (SD 35.66; *P*<.01)
- Time using app during trial: NS

**Ridad et al [44], 2020; Philippines**

- Participants showed significant improvement on all diabetes self-care activities
  Foot care: pretest score: 6.00 vs posttest score: 8.58 (*P*<.01)
  Diet: pretest score: 16.44 vs posttest score: 22.15 (*P*<.01)
  Physical activity: pretest score: 7.08 vs posttest score: 9.77 (*P*<.01)
  Blood sugar monitoring: pretest score: 0.42 vs posttest score: 5.58 (*P*<.01)
  Blood sugar level: pretest score: 2.13 vs posttest score: 1.67 (*P*<.01)
- Time using app during trial: NS

### Quality Assessment

As no study in First Nations Peoples from Australia was identified, the Aboriginal and Torres Strait Islander Quality Appraisal Tool was not used. Of the included studies, the randomized controlled trials (3/6, 50%) displayed a risk of bias of *some concerns*, as assessed by the RoB 2 tool (Table S1 in Multimedia Appendix 3). The risk of bias owing to randomization was rated as *some concerns* mainly because of a lack of data regarding whether the allocation sequence was concealed until after enrollment. All studies used random sequence generation and reported no baseline differences between groups (except for the study by Kilic and Karadağ [28], where previous diabetic foot education was higher in the control group). The risk of bias owing to deviations from the intended interventions was rated *low* in all studies. Only the study by Kilic and Karadağ [28] could be considered to have some bias as a result of missing outcome data, with >5% of the participants lost to follow-up and no analyses conducted to compare those lost to follow-up with the full sample. None of the participants in the trial intervention groups were blinded to their allocation because the mHealth app was installed on their mobile phones. This raises some concerns with certain outcome measures because the knowledge of the intervention may have influenced the participants’ self-reported outcomes (diabetes self-management and self-efficacy questionnaires as well as diabetes-related foot knowledge). However, 2 (67%) of the 3 randomized controlled trials also reported additional outcome measures (clinical outcomes) that were not likely to be affected by the participants’ knowledge of the intervention received [28,43].

The Health Evidence Bulletins Wales checklist was used to assess a qualitative study [42], a pilot study [44], and a validation study [27] (Table S2 in Multimedia Appendix 3). All studies (n=3) provided detailed information regarding study population, aims, and outcomes. Although the qualitative study [42] stated that the interviews were coded using thematic analysis, minimal information regarding the analysis was presented. No details were provided regarding the researchers’ role or background, whether the interview questions were predetermined, why both interviews and focus groups were used, how many people attended the focus groups, how themes and concepts were identified in the data, and whether the participants received the interview data for confirmation and feedback. The length of follow-up within the studies (<12 wk) meant that the longer-term impact of the mHealth apps on diabetes-related knowledge and self-care management is unknown. Because of these limitations, the findings of this study should be viewed with caution.

## Discussion

### Principal Findings

No study investigating an mHealth app for the assessment and management of diabetes-related foot health in First Nations Peoples from Australia was identified. Of the 6 included studies in non-Indigenous populations, 1 (17%) reported high levels of acceptability of the mHealth app content for self-care by people with diabetes and diabetes specialists [27]. The remaining studies (5/6, 83%) found that participants’ self-reported diabetes-related knowledge and self-management skills improved after the use of their mHealth app [26,28,42-44]. This suggests that, over the short term (<6 mo), the use of mHealth apps by patients with diabetes results in improved diabetes-related knowledge and self-care ability. However, further research is required to investigate whether these findings translate to the prevention of DFD and whether there is an improvement in outcomes such as a reduction in ulcer healing time or the rates of amputation.

In Australia, reducing the burden of DFD in First Nations Peoples, particularly those living in rural and remote areas, is a national priority [45]. This requires a multifaceted approach that navigates the obstacles of geographical distance and internet connectivity, facilitates effective communication among health professionals, integrates with existing services, engages the patients in effective preventive care strategies, and better aligns rural and remote service delivery with Australian best practice DFD prevention and management guidelines [46,47]. Although this systematic review did not find any studies using mHealth apps for the assessment and management of diabetes-related foot health in First Nations Peoples in Australia, mHealth apps have been used in working with these populations for other health conditions, including mental health and weight management, and may be a complementary method to help reduce the disproportionate burden of DFD experienced by First Nations Peoples [25]. However, the acceptability, design, implementation, and evaluation of mHealth apps must be Community-led, embrace a co-design approach, and feature a participatory action research approach [48]. mHealth apps must be culturally safe and therefore must actively engage with, and privilege, First Nations epistemologies and ontologies [49,50] (eg, locally produced mHealth strategies that privilege First Nations voices, incorporate culturally appropriate content as judged by local Communities, and promote engagement through alternative mediums to text such as graphics and animations that have been demonstrated to have the best chance of successful implementation [25]). It is through these approaches that First Nations Communities are empowered to take control and have ownership of their own health and well-being [48,51].

In non-Indigenous populations globally, our review has demonstrated short-term improvements in self-reported knowledge of, and self-care ability for, diabetes-related foot health with the use of an mHealth app. However, the impact of these interventions on adherence to routine foot health monitoring by health professionals and DFD occurrence and outcomes have not been evaluated. This is a key recommendation from international guidelines for the prevention and management of DFD [46]. Nevertheless, mHealth apps have been used to improve clinical health outcomes for a number of chronic conditions, including depression, obesity, alcohol dependence, and diabetes [25,52]. Previous studies, including those in patients with diabetes, have focused on mHealth apps that aim to improve glycemic control, with the outcomes from a meta-analysis supporting their use in people with type 1 diabetes [53]. These studies used features similar to those of the mHealth apps developed by the studies in this systematic review for improving knowledge and self-care management of DFD, including patient education resources [26-28,42-44], the ability for patients to input data [28,44], and reminder notifications [26,44]. These features may help improve uptake and engagement of the mHealth apps; however, it is important to consider that mHealth apps may need to use a variety of features to support different types of learners.

Previous studies support the need to use mHealth apps as part of a suite of measures, including face-to-face and telehealth care [54]. It is proposed that mHealth apps should aim to incorporate functions to increase patient access to health care services and interaction with health care professionals, such as the ability to schedule appointments via the app as well as support the coordination of health care through patient data sharing. This is particularly relevant to geographically remote regions where in-person care and care across many health professions requires assistance or is not possible [55]. Inputting clinical data in mHealth apps also supports information sharing, improves efficiency by reducing redundant assessments, and supports continuity of care within and among health care professions to help ensure a patient-centered approach to the assessment and management of chronic health conditions such as diabetes [56]. In addition, there is a growing body of evidence supporting the use of nonintrusive advanced analytics to evaluate user engagement with the mHealth app [57]. This is because limited engagement has been identified as a key reason for the poor performance of mHealth apps for the management of other medical conditions [57].

Our systematic review demonstrated improved self-management of foot health and increased self-care knowledge in non-Indigenous populations with diabetes after the use of an mHealth app. These outcomes support the use of mHealth apps as an effective health promotion strategy that can increase knowledge and self-care capacity. As demonstrated by international guidelines, evidence-based care is effective for reducing the complications associated with DFD, such as foot ulcer and amputation [11,58]. On the basis of the findings of our review, mHealth apps that incorporate evidence-based health promotion and prevention education, encourage patient engagement through patient-driven upload of data, and support the coordination of care delivery among health services are required to address the growing impact of DFD, particularly in priority populations and those living in rural and remote areas, including First Nations Communities. Such mHealth apps offer an innovative, sustainable, and long-term strategy that will work with, and for, discrete populations to help address health care delivery barriers relating to geographic distance and the coordination of care delivery from multiple health care providers.

### Limitations and Directions for Future Research

The results of this systematic review need to be considered in the context of a number of limitations. Although systematic searching included a number of First Nations databases and resources, it is important to note that First Nations research may be published in alternative forms to academic publications and will not have been identified in this search. The differences in study designs and interventions as well as the small number of included studies precluded a meta-analysis. Furthermore, the small number of participants in some of the included studies (4/6, 67%) as well as the homogeneous nature of participants (majority community-dwelling patients categorized as low risk from metropolitan or regional areas) reduce the ability of the findings of this review to be generalized to all people with diabetes and support the need for further research in other subpopulations with diabetes, including patients with DFD complications such as foot ulceration and amputation, as well as First Nations Peoples from Australia. Future research that aims to develop mHealth apps should consider using the assessment framework for mHealth apps [59] and learn from the findings from previous studies that have investigated barriers and facilitators to the use of digital health technologies, including mHealth apps, in other conditions in First Nations populations in geographically diverse locations [25,47,60]. In addition, an evaluation of internet access as a potential barrier to end-user engagement needs to be undertaken. In the absence of internet access, efforts must be made to provide content that is accessible offline and functionality that enables communication without the internet, including via SMS text messages. This may be the case in remote First Nations Communities in Australia where, despite the mobile phone use rate being as high as 43% [61], limitations with internet connectivity remain [47]. Although there were 3 (50%) randomized controlled trials included in the 6 studies in this systematic review, there were some concerns with the quality of their methodology, including a lack of blinding of participants to the intervention, but this is likely a limitation of the nature of this type of intervention. The knowledge and self-care management outcomes reported in these randomized controlled trials are therefore potentially biased and should be interpreted in the context of this limitation. The self-report nature of the knowledge and self-care management practice outcomes across all studies may be subject to recall bias. However, the impact of this may be limited owing to the integration of features such as on-demand education resources in all studies [26-28,42-44] and the ability to input data instantly within the mHealth apps in 2 (33%) [28,44] of the 6 included studies.

### Conclusions

This systematic review did not identify any studies that evaluated mHealth apps for the assessment and management of DFD in First Nations Peoples in Australia. The included studies (n=6) conducted in the general population in adults with relatively low risk with diabetes support the use of mHealth apps to improve diabetes-related knowledge and self-care management. No data evaluating DFD outcomes were retrieved. Future research needs to focus on mHealth apps for populations for whom there is inadequate or ineffective service delivery and aim to evaluate the direct effects of mHealth apps on DFD outcomes. Future research needs to be for First Nations Peoples, without cross-cultural generalization, and for those living in geographically remote areas.

## Appendix

Multimedia Appendix 1 Electronic database search strategy.

Multimedia Appendix 2 Population, intervention, comparison, and outcomes.

Multimedia Appendix 3 Quality appraisal of included studies.

Multimedia Appendix 4 PRISMA checklist.

## Abbreviations

DFD: diabetes-related foot disease
MeSH: Medical Subject Headings
mHealth: mobile health
PICO: population, intervention, comparison, and outcomes
PRISMA: Preferred Reporting Items for Systematic Reviews and Meta-Analyses
RoB 2: revised Cochrane risk-of-bias tool for randomized trials
