# Supplementary material for: The Use of mHealth Apps for the Assessment and Management of Diabetes-Related Foot Health Outcomes: Systematic Review
Source: J Med Internet Res. 2023 Oct 4;25:e47608. doi: 10.2196/47608 (PMC10585435; doi:10.2196/47608)
Supplement: Multimedia Appendix 1 [file jmir_v25i1e47608_app1.docx]

**Multimedia Appendix 1.** Electronic database search strategy.

First Nations specific search strategy

PubMed

("australia"[MeSH Terms] OR "australia*"[Title/Abstract]) AND ("health services, indigenous"[MeSH Terms] OR ("native hawaiian or other pacific islander"[MeSH Terms] OR ("native"[All Fields] AND "hawaiian"[All Fields] AND "or"[All Fields] AND "Other"[All Fields] AND "pacific"[All Fields] AND "islander"[All Fields]) OR "native hawaiian or other pacific islander"[All Fields] OR ("native"[All Fields] AND "hawaiian"[All Fields]) OR "native hawaiian"[All Fields]) OR ("Other"[All Fields] AND "native hawaiian or other pacific islander"[MeSH Terms]))

AND

"diabetic foot"[MeSH Terms] OR "foot ulcer"[MeSH Terms] OR (("diabet*"[All Fields] AND "n8"[All Fields]) AND ("foot"[Title/Abstract] OR "feet"[Title/Abstract] OR "wound"[Title/Abstract] OR "ulcer*"[Title/Abstract] OR "lower limb"[Title/Abstract]))

Informit Indigenous Collection databases

All Fields: diabet* AND [All Fields: foot OR All Fields: feet OR All Fields: 'lower limb' OR All Fields: wound OR All Fields: ulcer]

MEDLINE/Embase search strategy

1. exp diabetic foot/

2. exp Foot Ulcer/

3. (diabet* adj8 (foot or feet or wound or ulcer* or 'lower limb')).tw.

4. 1 or 2 or 3

5. Smartphone.mp.

6. Smart-phone.mp.

7. ((mobile or cell) adj2 phone*).mp.

8. iphone.mp.

9. android.mp.

10. ipad.mp.

11. 'mobile application*'.mp.

12. 'mobile app*'.mp.

13. mobile applications/

14. mhealth.mp.

15. exp cell phones/

16. (monitor* adj4 (home or remote)).mp.

17. 5 or 6 or 7 or 8 or 9 or 10 or 11 or 12 or 13 or 14 or 15 or 16

18. 4 and 17

CINAHL

( TITLE-ABS-KEY ( ( smartphone*  OR  smart-phone* )  OR  ( ( mobile  OR  cell )  W/2  phone* )  OR  ( iphone  OR  android  OR  ipad )  OR  ( 'mobile  AND application*'  OR  'mobile  AND app*'  OR  mhealth )  OR  ( monitor*  W/4  ( home  OR  remote ) ) ) )  AND  ( TITLE-ABS-KEY ( diabet*  AND  ( foot  OR  feet  OR  wound  OR  ulcer*  OR  {lower limb} ) ) ) Limiters - Exclude MEDLINE records

SCOPUS

( TITLE-ABS-KEY ( ( smartphone*  OR  smart-phone* )  OR  ( ( mobile  OR  cell )  W/2  phone* )  OR  ( iphone  OR  android  OR  ipad )  OR  ( 'mobile  AND application*'  OR  'mobile  AND app*'  OR  mhealth )  OR  ( monitor*  W/4  ( home  OR  remote ) ) ) )  AND  ( TITLE-ABS-KEY ( diabet*  AND  ( foot  OR  feet  OR  wound  OR  ulcer*  OR  {lower limb} ) ) )
