# Supplementary material for: The Use of mHealth Apps for the Assessment and Management of Diabetes-Related Foot Health Outcomes: Systematic Review
Source: J Med Internet Res. 2023 Oct 4;25:e47608. doi: 10.2196/47608 (PMC10585435; doi:10.2196/47608)
Supplement: Multimedia Appendix 2 [file jmir_v25i1e47608_app2.docx]

**Multimedia Appendix 2.** PICOs

Randomised controlled trials:

-The population is First Nations Peoples in Australia and non-Indigenous populations globally with diabetes-related disease

-The intervention is mHealth applications

-The control is a placebo, non-intervention, waitlist control, or equivalent

-The outcome could include qualitative or quantitative evaluation of the mHealth application such as the usability, acceptability, feasibility, or effect on diabetes-related foot health outcomes

Observational or prospective studies:

-The population is First Nations Peoples in Australia and non-Indigenous populations globally with diabetes-related disease

-The intervention is mHealth applications

-There is no control due to the nature of the study types

-The outcome could include qualitative or quantitative evaluation of the mHealth application such as the usability, acceptability, feasibility, or effect on diabetes-related foot health outcomes
